# Supplementary material for: Genomic analysis of Mycobacterium brumae sustains its nonpathogenic and immunogenic phenotype
Source: Front Microbiol. 2023 Jan 5;13:982679. doi: 10.3389/fmicb.2022.982679 (PMC9850167; doi:10.3389/fmicb.2022.982679)

Supplementary Figure S3. Phylogenetic relationship of the available *M. brumae* genomic sequences. A) Maximum likelihood phylogenetic tree based on an alignment of the core-genome with a length of 293,792 bp and including *M. fallax* as outgroup. GTR model was used with bootstrap confidence of 1000 replicates. B) Zoom-in to the *M. brumae* cluster of the previous tree showed in A. Red dots indicate genomic sequences of the type strain (same as ATCC 51384).

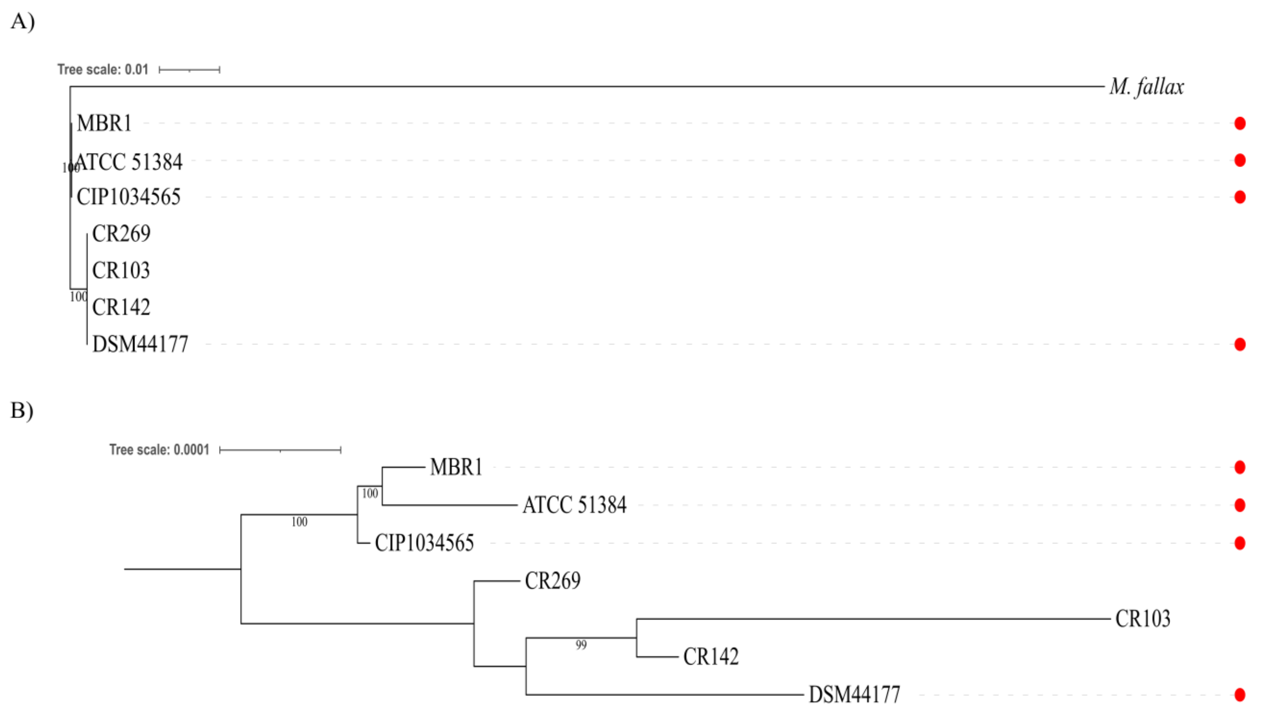

Supplement: Supplementary file 9 [file Image_1.PDF]
