# Supplementary material for: Genomic analysis of Mycobacterium brumae sustains its nonpathogenic and immunogenic phenotype
Source: Front Microbiol. 2023 Jan 5;13:982679. doi: 10.3389/fmicb.2022.982679 (PMC9850167; doi:10.3389/fmicb.2022.982679)
Supplement: Supplementary file 11 [file Image_3.PDF]

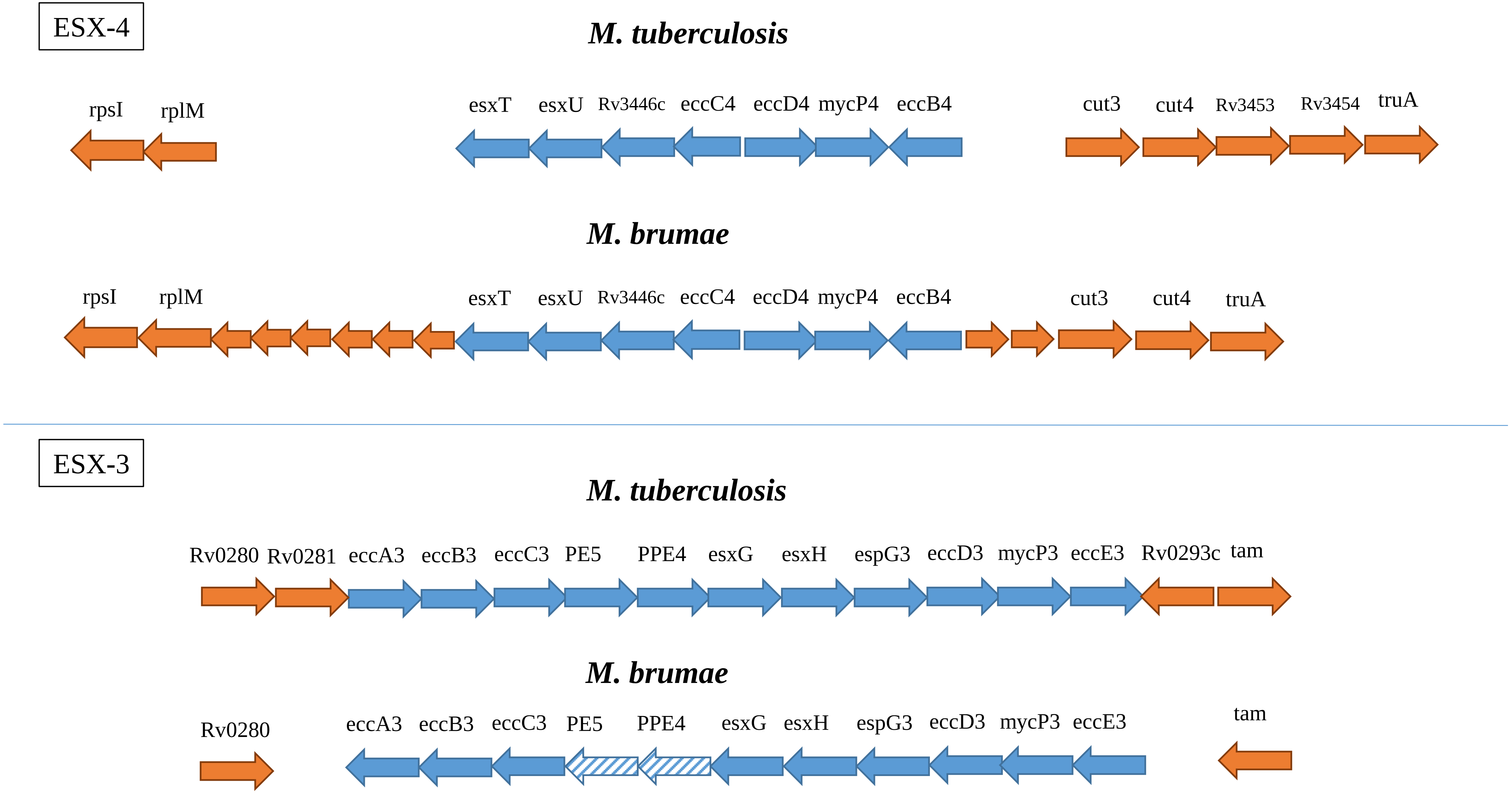

Supplementary Figure S3. Comparison of the ESX-3 and ESX-4 clusters in *M. tuberculosis* H37Rv and *M. brumae*. Genes from the cluster are colored in blue and flanquing genes are colored in orange. Stripped arrows indicate the genes found with low similitude (under 70% protein identity).
