# Supplementary material for: Genomic analysis of Mycobacterium brumae sustains its nonpathogenic and immunogenic phenotype
Source: Front Microbiol. 2023 Jan 5;13:982679. doi: 10.3389/fmicb.2022.982679 (PMC9850167; doi:10.3389/fmicb.2022.982679)
Supplement: Supplementary file 13 [file Image_5.PDF]

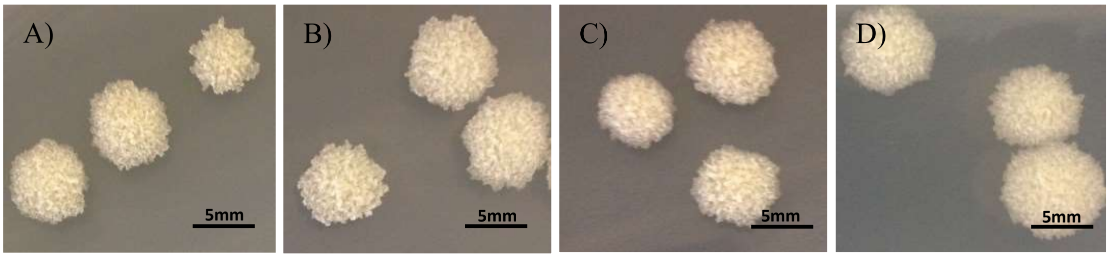

Supplementary Figure S2. Colonies of CR270 (A), CR269 (B), CR142 (C) and CR103 (D) *M. brumae* strains grown on Middlebrook 7H10 medium for one week. Pictures were taken with Nikon camera.
